# Antisense RNA therapies for muscular dystrophies

**DOI:** 10.1177/22143602251324858

**Published:** 2025-03-27

**Authors:** Virginia Arechavala-Gomeza, Andrea López-Martínez, Annemieke Aartsma-Rus

**Affiliations:** 1 Nucleic Acid Therapeutics for Rare Diseases (NAT-RD), Biobizkaia Health Research Institute, Barakaldo, Spain; 2 Ikerbasque, Basque Foundation for Science, Bilbao, Spain; 3 Department of Human Genetics, Leiden University Medical Center, Leiden, the Netherlands

**Keywords:** antisense oligonucleotide, siRNA, muscle delivery, muscular dystrophy

## Abstract

Inherited muscular dystrophies are a heterogeneous group of diseases, caused by different types of genetic mutations. RNA therapies, and particularly antisense oligonucleotides, offer a palette of therapeutic strategies to either reduce the production of harmful proteins or to restore or increase protein expression. Consequently, they offer therapeutic promise for multiple forms of muscular dystrophies. This review outlines the different RNA therapy types considered for the treatment of Duchenne muscular dystrophy, facioscapulohumeral muscular dystrophy and myotonic dystrophy, emphasizing the strategies used to deliver these therapies to skeletal muscle with a focus on approaches that have reached the clinical trial stage.

## Introduction

Inherited muscular dystrophies are a heterogeneous group of diseases that are characterised by the progressive loss of muscle tissue and function.^1^ These conditions have different types of inheritance, such as X-linked recessive for Duchenne muscular dystrophy (DMD), autosomal recessive for various forms of limb-girdle muscular dystrophy and autosomal dominant for facioscapulohumoral muscular dystrophy (FSHD) and myotonic dystrophy (DM). While recessive diseases are caused by loss of function variants, dominantly inherited diseases are caused by toxic gains of transcript or protein function (DM and FSHD, respectively).

RNA therapeutics target the transcript level, offering means to reduce the production of toxic transcripts or proteins, or to restore protein production.^2^ As such, these approaches offer therapeutic opportunities for multiple inherited muscular dystrophies.

This review will outline the various types of RNA therapeutics that can be utilised to treat different inherited muscular dystrophies. A key consideration for therapeutic success is achieving sufficient delivery to the target tissue,^3,4^ namely skeletal muscle, and we will outline different strategies to enhance this process. We will focus on approaches that are currently undergoing or nearing clinical trials, with some insights drawn from past unsuccessful developments, where relevant.

## RNA therapy modalities for inherited muscular dystrophies

Antisense oligonucleotides (ASOs) are either single or double-stranded DNA or RNA analogues. They require chemical modifications to achieve drug-like properties, such as enhancing resistance against nucleases, reducing the inflammatory response prompted by the foreign nature of DNA and RNA, and increasing bioavailability.^2^ The most common modification is the phosphorothioate (PS) backbone, which increases bioavailability of single-stranded ASOs by enabling low-affinity binding with serum proteins, thus preventing renal clearance.^5^ Additionally, PS backbones enhance transmembrane uptake. However, since the PS backbone reduces the affinity for RNA transcripts, additional modifications to the ribose have been developed. The 2′*O*-methyl (2OMe) and 2′*O*-methoxyethyl (MOE) RNA ribose modifications are the most used to increase the affinity of the ASO for the transcript while reducing the inflammatory response and other side effects that can happen in ASOs with only a PS modification.

In this review, we will focus on antisense RNA therapies, i.e., synthetic antisense oligonucleotides (ASO) that target RNA transcripts via Watson-Crick base pairing in order to bring about a treatment effect, as these are currently in or close to clinical trial applications for muscular dystrophies. Basically, the RNA therapies discussed here act in two different ways: by inducing cleavage of the targeted transcript or by steric hindrance to prevent binding of proteins to the transcript, e.g., to modulate splicing. Which modality to use will depend on the pathomechanism of the disease.

A discourse on the plethora of chemical modifications available is beyond the scope of this review and we refer the interested reader to a recent expert review.^6^ However, it is important to bear in mind that the modifications that can be used depend on the mechanism of action of the ASOs. Generally speaking, ASOs that induce cleavage of target transcripts have less room for modifications as they trigger cellular mechanisms that do not tolerate (too many) modifications. In contrast, ASOs that act via steric hindrance have more flexibility for modifications as they must bind the transcript with sufficient affinity to block or displace proteins.^2^

Reduction of transcript expression can be achieved by single-stranded ASO and double-stranded siRNAs. ASOs will trigger RNase H, an enzyme that cleaves RNA-DNA hybrids. RNase H cleaves the 2′-O- position of the ASO, meaning that modifications of the sugar are not well tolerated. However, it is not desirable to have an ASO with only a PS modification due to safety issues and a limited target affinity. Thus, RNase H inducing ASOs have a gapmer construction where the centre will only have a PS modification to facilitate RNase H cleavage, while the flanks are also sugar modified to ensure high affinity binding to the transcript.^6^

siRNAs are double-stranded ASOs that hijack the RNA-induced silencing complex (RISC), a system present in every cell that incorporates miRNAs, to modulate expression levels of transcripts. The goal of siRNA therapies is to trick RISC into accepting a sequence of choice into the complex, so that a target transcript is cleaved by RISC. The double-stranded siRNA has one strand that must enter theRISC and target the transcript (the guide strand), and a sense or passenger strand. Chemical modifications of the guide strand are minimally tolerated, as this strand has to be accepted by RISC, but the passenger strand is more tolerant to chemical modifications. Notably, chemical modifications of the passenger strand also serve to prevent it from being incorporated into the RISC complex, as this could lead to unintended effects.^6^

Single-stranded ASOs can also be used for steric hindrance. This was originally used to block the translation of transcripts. However, to achieve this, high-affinity ASOs are needed, as the ribosomal machinery would otherwise remove the ASO during translation. Ryszard Kole and colleagues attempted to use steric hindrance ASOs to modulate splicing, the process by which pre-mRNA is converted into mRNA by removing introns and joining exons.^7^ Splice modulation can be used to skip an exon from the mRNA or to increase the inclusion of an exon into the mRNA. As mentioned above, these ASOs can be heavily chemically modified. The most commonly used modification for splice-modulating ASOs is the phosphorodiamidate morpholino oligomer (PMO), which has little resemblance to DNA, and as such is not recognised by nucleases, but which still binds to RNA via Watson-Crick base pairing.^8^

## Applying ASOs to muscular dystrophies

This section explains the rationale for using ASOs to treat DMD, DM1 and FSHD and reviews the state of the art for unconjugated ASOs.

### Duchenne muscular dystrophy

DMD is caused by mutations, mostly deletions involving one or more exons in the *DMD* gene, which prevent the production of dystrophin, a protein that connects the actin cytoskeleton to the extracellular matrix in muscle fibers.^9^ In the absence of dystrophin, muscles are susceptiblee to contraction-induced injury, leading to chronic accumulation of damage, inflammation and eventual failure to regenerate. Over time, muscle tissue is replaced by fibrotic and adipose tissue and muscle function is progressively lost. Patients present with the first symptoms before the age of 4 years, usually lose ambulation before the age of 12 years and then progressively lose arm function, develop respiratory insufficiency requiring assisted ventilation and cardiomyopathy. With multidisciplinary management of symptoms and treatment with glucocorticosteroids, patients can live into their 3^rd^ or 4^th^ decade, but there is a clear unmet medical need.^9^

As the disease is caused by the lack of dystrophin, restoration of dystrophin is anticipated to have therapeutic effects. However, traditional gene addition therapy is challenging because the size of the dystrophin mRNA transcript exceeds of the size of the only viral vector currently capable of transducing skeletal muscle, i.e., the adeno-associated viral vector (AAV).^10^ Instead, gene addition therapy for DMD uses micro-dystrophin genes, that encode engineered short dystrophins that contain the key domains to maintain the linker function and thus hopefully slow down disease progression. The micro-dystrophins are expected to be partially functional, as patients with Becker muscular dystrophy (BMD) have dystrophins that are internally deleted but maintain the crucial domains for the linker function. BMD-type dystrophins are much larger than micro-dystrophins and are expected to be more functional, but their transcript length is too large for AAV.

**Figure 1.**
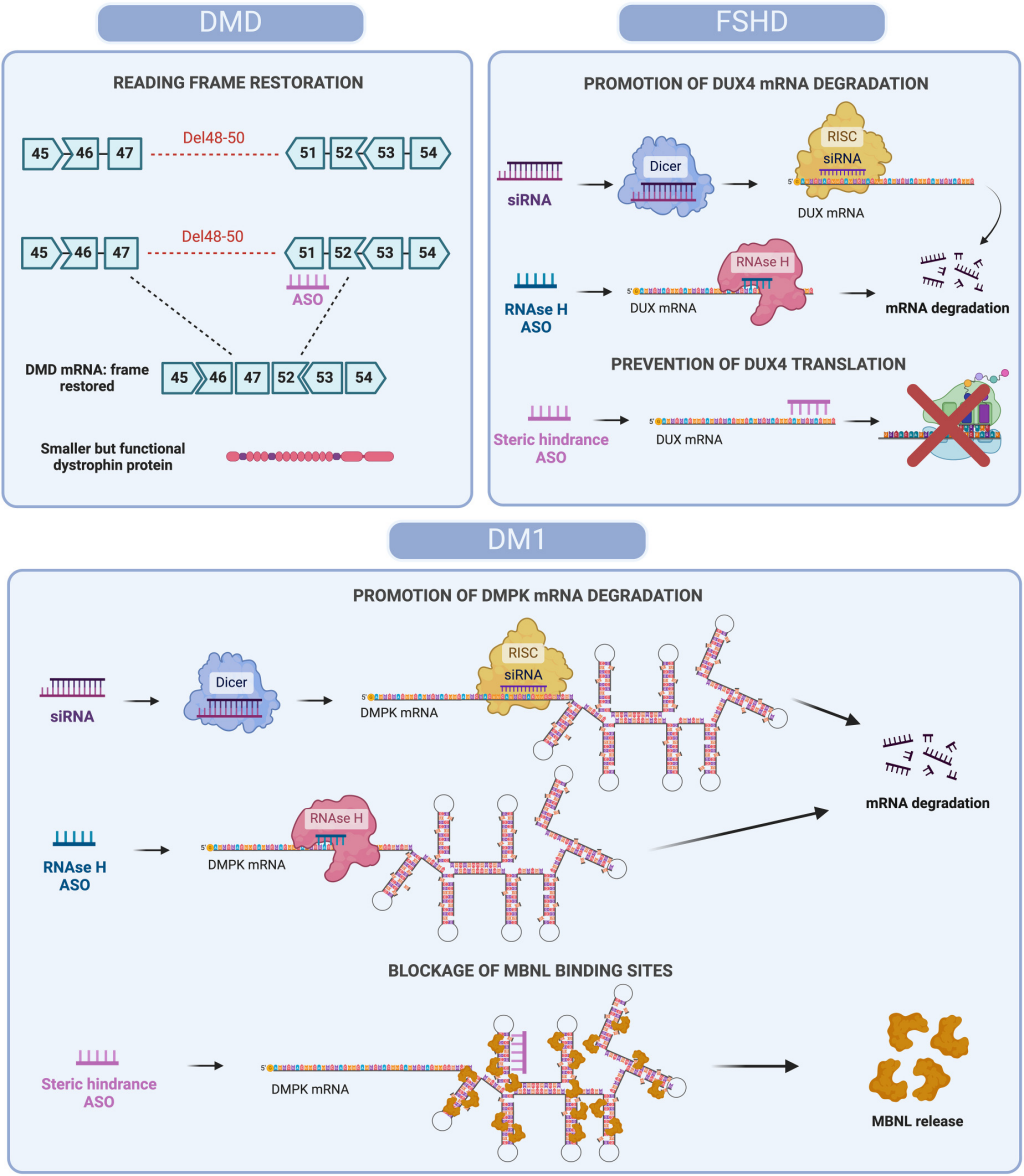
Antisense oligonucleotide-based strategies for mRNA modulation in DMD, FSHD and DM1. Different ASOs-based approaches to restore protein function or reduce toxicity are represented. In DMD, steric hindrance ASOs skip specific exons (e.g., Del48-50) to restore the DMD mRNA reading frame, allowing production of a shorter but functional dystrophin protein. In FSHD, siRNAs and RNase H ASOs degrade DUX4 mRNA, while steric hindrance ASOs prevent translation, reducing toxic DUX4 protein levels. In DM1, siRNAs and RNase H ASOs degrade expanded DMPK mRNA, while steric hindrance ASOs block MBNL binding, releasing MBNL proteins and restoring splicing function. Created with BioRender.com.

However, ASOs offer the possibility of allowing patients with DMD to produce BMD-type dystrophins, by modulating the pre-mRNA processing of dystrophin transcripts. ASOs can hide target exons from the splicing machinery, so that the exon is not included (skipped) in the mRNA. Counter-intuitively, making the deletion one exon larger, can restore the open reading frame, thus allowing the production of a BMD-like type of dystrophin rather than no dystrophin (Figure 1).^11,12^ It is important to note that the exon skipping approach is mutation specific and an ASO targeting a specific exon will only apply to a subset of patients. Due to the clustering of deletions in a hotspot region between exons 42 and 55, skipping of certain exons will apply to relatively large groups (4–14%), while skipping other exons will apply to very small groups of less than 0.5%.^13^

Proof of concept for ASO-mediated exon skipping and dystrophin restoration has been established in patient-derived cells and animal models for both the 2OMePS and PMO chemistry.^11,12,14,15^ This showed that most of the dystrophin exons can be efficiently skipped. However, clinical development has focused primarily on exon 51 skipping, as this applied to the largest group of patients (14%). Both 2OMePS (drisapersen) and PMO (eteplirsen) ASOs were tested in clinical trials.^16–19^ Drisapersen was tested in various clinical trials, where weekly subcutaneous treatment of 6 mg/kg could increase dystrophin levels. Side effects were observed, including injection site reactions, proteinuria and in a small percentage thrombocytopenia.^18,19^ Functionally, in younger patients, treatment appeared to slow down disease progression in 24 and 48-week placebo-controlled phase 2 trials. However, in a larger placebo-controlled phase 3 trial involving 188 patients treated for 48 weeks, the primary endpoint (distance walked in 6 min, 6MWD) was not met.^18^ The sponsors (BioMarin/Prosensa) sought to get FDA approval based on post-hoc analyses showing a difference in 6MWD in a subgroup of patients, but this was not granted.^20^

Eteplirsen was tested in a smaller group of patients, ultimately testing a dose of 30 mg/kg and 50 mg/kg in 12 patients in an open label study.^17,21^ Side effects were primarily related to the weely intravenous infusions.^22^ Restoration of dystrophin was seen in each of the treated patients, although the increases were very minimal (<0.5%). Due to the open label nature of the trial, patient function is compared to natural history cohorts.^22^ The FDA granted eteplirsen accelerated approval in 2016 based on the dystrophin restoration data,^23^ but insisted that additional clinical trials to confirm functional effects had to be collected by the sponsor (Sarepta).^20,24^ The confirmatory study, in which patients receive weekly treatment with eteplirsen or placebo for 3 years, is currently still ongoing. Meanwhile, additional PMOs targeting other *DMD* exons have been approved based on dystrophin restoration: golodirsen (targeting exon 53, developed by Sarepta), casimersen (targeting exon 45, developed by Sarepta) and viltolarsen (targeting exon 53, developed by NS Pharma).^25^ For these PMOs dystrophin restoration levels are still very modest, varying between 1 and 5%. A clinical trial to evaluate a PMO targeting exon 44 (brigadirsen) is done by NS Pharma. Weekly intravenous dosing with 40 or 80 mg/kg for 24 weeks resulted in an expression of 16.6% and 24.5% dystrophin, respectively. However, at baseline patient already expressed ∼7% of dystrophin. Notably, these results for now are preliminary as each dose was tested in cohorts over only 3 patients per dose.

### Myotonic dystrophy type 1

DM1 is a multisystemic disorder, caused by CTG repeat expansions in the 3′ UTR of the *DMPK* gene.^26^ Once the expansion exceeds a pathological threshold, it becomes unstable somatically and also in germ cells. Consequently, there is anticipation: offspring of DM1 patients who inherit the expansion tend to have a longer expansion than their parents, leading to an earlier onset and more severe disease.

The transcripts from the expanded *DMPK* gene will form stable secondary and tertiary structures that sequester proteins, mainly those that bind to CUG motifs in the nucleus, such as muscleblind-like protein family (MBNL). This prevents these proteins from performing their normal function of modulating alternative splicing regulation of various transcripts and consequently, many transcripts are misspliced in DM1 patients.^27^ This results in wide range of symptoms including myotonia, muscle wasting and weakness, cataracts, insulin resistance, cardiac conduction problems, daytime sleepiness, and gastrointestinal problems. The type and severity of symptoms vary between patients.^26^

For some symptoms there is a direct relation with the missplicing of a specific gene transcript. For example, missplicing of the chloride channel (*CLCN1*) in skeletal muscle tissue is responsible for the myotonia as, due to the deregulation of splicing factors, an exon is included in the CLCN1 transcripts that is normally only included in the transcripts during foetal development. An ASO targeting this exon can restore the production of the postnatal CLCN1 transcripts. Treatment of DM1 mouse models with this ASO has been shown to reduce myotonia.^28^ However, such a transcript-by-transcript approach is cumbersome and also challenging because many symptoms are not clearly linked to the missplicing of a single transcript. It is therefore more pragmatic to target the root cause of the pathology, i.e., the sequestration of splicing factors by the expanded transcripts.

Different types of approaches have been used for this (Figure 1). First, a (CAG)_7_ ASO targeting the CUG repeat was tested.^29^ This may appear to be an aspecific approach, as this ASO would target all transcripts with a CUG repeat, including the DMPK transcript produced by the unaffected allele. However, as the CUG repeat in the *DMPK* gene of DM1 patients can be hundreds or thousands of repeats long, the ASO will primarily bind to the DMPK transcript with the expanded allele due to this overabundance. The hypothesis is that ASO binding will resolve the repeat structures, releasing the splicing factors. Studies with these ASOs have indeed shown restoration of normal splicing, but also reduced expression of the expanded repeats via an unknown mechanism. It is possible that the ‘unstructuring’ makes the transcripts available for degradation.

A second approach used RNase H ASOs targeting DMPK transcripts.^30^ This approach is not allele specific, and therefore reduces DMPK in general. However, it is expected to be well tolerated postnatally. In studies in animal models this approach reduced DMPK transcript levels, normalised missplicing and improved histology.^30^ This ASO (baliforsen) was also tested in a clinical trial, but its development was stopped as delivery of ASOs to skeletal muscle was insufficient to expect therapeutic effects.^31^

### Facioscapulohumeral muscular dystrophy

FSHD is characterised by muscle weakness in the facial, shoulder and leg muscles. Age of onset can occur in childhood, midlife or later, and the severity is variable.^1^ The weakness, often, starts asymmetrically. FSHD is caused by the expression of DUX4 in skeletal muscle, which is toxic postnatally.^32^ The *DUX4* gene is located in the subtelomeric region of chromosome 4, in a macrosatellite repeat and is normally silenced postnatally by epigenetic silencing. However, in individuals with a repeat contraction, the gene can be activated and, provided the individual carries a permissive allele containing a polyadenylation signal that allows the production of a stable DUX4 transcript, this enables the production of DUX4 protein.^32^ This triggers several pathological pathways, leading to muscle damage, inflammation and eventually loss of muscle tissue.

Since DUX4 expression is the trigger of pathology, reducing or preventing DUX4 expression by ASOs would be expected to have therapeutic effects. Several approaches have been tested: ASOs that prevent polyadenylation or DUX4 splicing via steric hindrance, or ASOs that induce RNase H-mediated cleavage of DUX4 transcripts. *In vitro*, this resulted in reduced expression of DUX4 and its target genes, and improved atrophy.^33–36^ ASO treatment was able to reduce the expression of DUX4 and DUX4 target genes in muscle explants from FSHD patientsin mice.^36^ Finally, long-term systemic treatment of an inducible FSHD mouse model with ASOs targeting the DUX4 polyadenylation signal reduced expression of DUX4 and its target genes, improved muscle mass and function, and reduced collagen deposition.^37^

## How to improve delivery of ASOs to skeletal muscle

**Figure 2.**
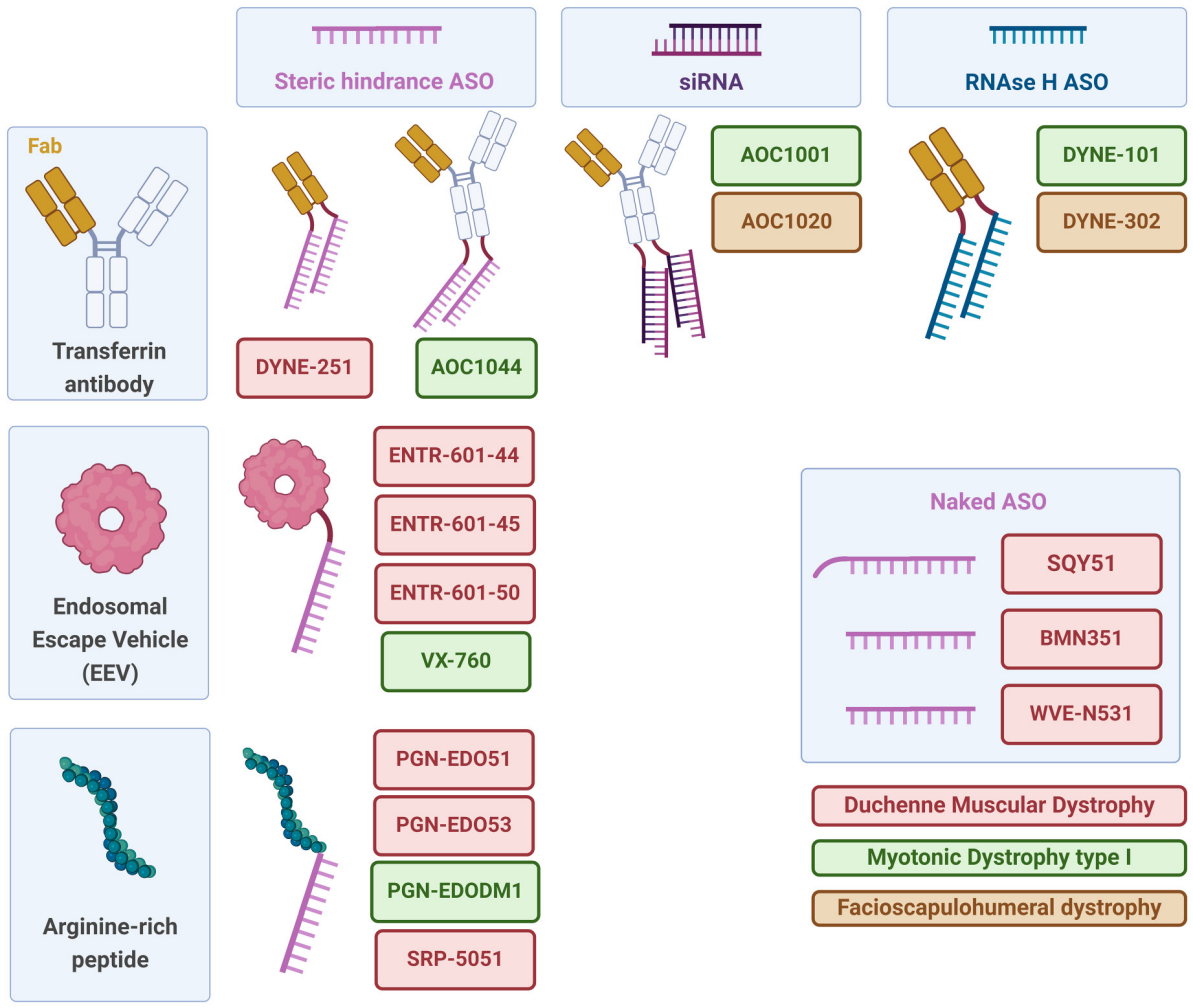
Delivery strategies tested for antisense oligonucleotide-based therapies in DMD, DM1 and FSHD. Various delivery systems and oligonucleotide types used to target DMD, DM1 and FSHD are represented. Delivery vehicles, such as transferrin antibodies, endosomal escape vehicles, and arginine-rich peptides, enhance the cellular uptake and delivery efficiency of oligonucleotides. Antisense oligonucleotide-based strategies include steric hindrance ASOs, which block translation or abnormal RNA structures, siRNAs, which promote mRNA degradation, and RNase H-dependent ASOs, which induce RNA degradation via RNase H. Lastly, naked ASOs, or ASOs with a palmitic acid tail, which are delivered without carriers, are represented. Created with BioRender.com.

In summary, based on proof-of-concept research in cultured cells and animal models, ASOs have the potential to address the various diseases in principle. However, the bottleneck for clinical application is the insufficient delivery of ASOs to the skeletal muscle. It is known that dystrophic muscle takes up more ASO than healthy muscle, thus giving DMD and FSHD an advantage over DM1, where there is no dystrophic pathology.^38,39^ Nevertheless, it is clear that even for dystrophic muscle, there is room for improvement based on the very low levels of dystrophin restoration in DMD with high, weekly, intravenous doses of PMOs. There are several ways by which attempts are being made to improve the efficacy of ASOs for muscular dystrophies. An overview of approaches at or near the clinical trial stage can be found in Table 1 and Figure 2. It is noteworthy that most of the trial results have only been reported in press releases and not in peer-reviewed publications (provided as links to the press release to make the distinction with publications clear). In addition, for DMD exon skipping approaches, it should be taken into account that accurate measurement of low dystrophin levels is challenging^40^ and that dystrophin levels reported by different sponsors may not always be comparable.

**Table 1.** Approaches to improve antisense therapies for muscular dystrophies moving to or in a clinical setting.

Compound (Sponsor)	Disease	Approach	Clinicaltrial.gov number
AOC1001 (Avidity Biosciences)	DM1	Transferrin-receptor antibody conjugated to an siRNA targeting DMPK	NCT05027269
AOC1020 (Avidity Biosciences)	FSHD	Transferrin-receptor antibody conjugated to an siRNA targeting DUX4	NCT05747924
AOC1044/ delpacibart zotadirsen (Avidity Biosciences)	DMD (exon 44)	Transferrin-receptor antibody conjugated to a PMO targeting dystrophin exon 44	NCT05670730
BMN351 (Biomarin Pharmaceuticals)	DMD (exon 51)	Chemical modifications of an unconjugated ASO targeting dystrophin exon 51	NCT06280209
Brogodirsen (NS Pharma)	DMD (exon 44)	PMO ASO	NCT04129294
Dyne-101 (Dyne Therapeutics)	DM1	Transferrin-receptor antibody fragment conjugated to an RNase H antisense oligonucleotide targeting DMPK	NCT05481879
Dyne-251 (Dyne Therapeutics)	DMD (exon 51)	Transferrin-receptor antibody fragment conjugated to a PMO targeting dystrophin exon 51	NCT05524883
Dyne-302 (Dyne Therapeutics)	FSHD	Transferrin-receptor antibody fragment conjugated to an RNase H antisense oligonucleotide targeting DUX4.	Preclinical stage
ENTR-601-44 (Entrada Therapeutics)	DMD (exon 44)	Endosomal escape vehicle (EEV) conjugated to a PMO targeting dystrophin exon 44	None, trial ongoing in UK
ENTR-601-45 (Entrada Therapeutics)	DMD (exon 45)	EEV conjugated to a PMO targeting dystrophin exon 45	Preclinical stage
ENTR-601-50 (Entrada Therapeutics)	DMD (exon 50)	EEV conjugated to a PMO targeting dystrophin exon 50	Preclinical stage
PGN-EDO51 (Pepgen)	DMD (exon 51)	Arginine-rich peptide conjugated to a PMO targeting dystrophin exon 51	NCT06079736 (on hold since Dec 2024)
PGN-EDO53 (Pepgen)	DMD (exon 53)	Arginine-rich peptide conjugated to a PMO targeting dystrophin exon 53	Preclinical stage
PGN-EDODM1 (Pepgen)	DM1	Arginine-rich peptide conjugated to a PMO targeting the expanded CUG repeat in DMPK	NCT06204809
SRP-5051 / vesleteplirsen (Sarepta Therapeutics)	DMD (exon 51)	Arginine-rich peptide conjugated to a PMO targeting dystrophin exon 51	NCT04004065 (clinical development stopped Nov 2024)
SQY51 (Synthena Therapeutics)	DMD (exon 51)	Tricyclo DNA oligonucleotide with a palmitoyl lipid moeity conjugate targeting exon 51	NCT05753462
VX-760 (Vertex)	DM1	EEV conjugated to an antisense oligonucleotide targeting the expanded CUG repeat in DMPK	NCT06185764
WVE-N531 (Wave Life Sciences)	DMD (exon 53)	Chemically modified, stereopure antisense oligonucleotide targeting dystrohin exon 53	NCT04906460

### Chemical modifications

Chemical modifications can be used to improve the bioavailability of ASOs, increasing the likelihood of uptake of the ASO before being excreted. They can also increase the affinity of the ASO for the target transcript, thus improving the efficiency once the ASO is in the target cell. As mentioned above, steric hindrance ASOs in particular allow for extensive modification, so it is not surprising that this approach is currently only used for DMD exon skipping. Biomarin has developed a new exon 51 skipping ASO with additional modifications to the sugar and the backbone. In preclinical studies in a humanised mouse model, this ASO resulted in extensive exon skipping and dystrophin restoration and improved functional deficits.^41^ A clinical trial is currently underway to evaluate the efficiency of this ASO in eligible DMD patients.

Wave Life Sciences is developing an ASO for exon 53 skipping, where they are also using chemical modifications of the nucleotides and the backbone. In addition, they are using stereopure ASOs; the PS modification will lead to the formation of stereoisomers, i.e., it is possible to have asymmetric bonds that have slightly different properties in terms of affinity and stability. Since these isomers occur at each PS bond, ASOs are actually a mixture of 2^nth^ different isomers, where n is the number of PS bonds. By strategically placing of either left or right stereoisomer bonds, Wave aims to improve the efficiency of exon skipping ASOs.^42^ Previously, clinical development of a stereopure exon 51 skipping ASO (suvodirsen) was halted when no increase in dystrophin could be shown in treated patients (https://www.globenewswire.com/news-release/2019/12/16/1960830/0/en/Wave-Life-Sciences-Announces-Discontinuation-of-Suvodirsen-Development-for-Duchenne-Muscular-Dystrophy.html). An exon 53 skipping ASO is currently being tested in DMD patients, where initial results show that exon 53 skipping occurs in muscle biopsies after 12 weeks of treatment, although dystrophin levels were below the lower limit of quantification (https://ir.wavelifesciences.com/news-releases/news-release-details/wave-life-sciences-announces-upcoming-presentations-mda).

Finally, SQY therapeutics is developing tricyclo DNA (tcDNA) ASOs. These ASOs are fully chemically modified but have a phosphodiester backbone (so no stereoisomers occur). To facilitate bioavailability a palmitic acid tail is added. After preclinical optimization in the *mdx* mouse model,^43^ a tcDNA targeting exon 51 is now tested in a clinical trial.

### Cell penetrating peptides to improve delivery in general

It has been shown that positively charged peptides can increase uptake in a tissue-specific manner. Conjugation of these peptides can be used to improve the delivery of ASOs.^44,45^ Notably, this approach is only feasible for ASOs that are not negatively charged, such as PMOs. Several arginine-rich peptides conjugated to PMOs (pPMOs) are currently being tested in clinical trials. Sarepta is assessing a peptide conjugated version of eteplirsen (vesleteplirsen) in DMD patients at a monthly dose of 30 mg/kg. This resulted in higher dystrophin restoration than eteplirsen (6.5%) after 12 weeks of treatment, although hypomagnesemia, which could be managed with magnesium supplementation, was observed in some patients (https://investorrelations.sarepta.com/news-releases/news-release-details/sarepta-therapeutics-reports-positive-clinical-results-phase-2). However, recently Sarepta announced that they will stop the clinical development of vesleteplirsen due to safety concerns, related to irreversible kidney toxicity (https://www.parentprojectmd.org/sarepta-announces-discontinuation-of-srp-5051-development-for-duchenne/).

Pepgen also has an exon 51 targeting pPMO in clinical development in DMD patients. Here at the lowest dose of 5 mg/kg, dystrophin levels increased by 0.7% compared to the baseline after 4 monthly injections (https://investors.pepgen.com/news-releases/news-release-details/pepgen-announces-positive-data-low-dose-cohort-pgn-edo51-ongoing). No hypomagnesemia was reported in these patients. However, hypomagnesemia had previously been observed with this compound in a healthy volunteer study (https://investors.pepgen.com/news-releases/news-release-details/pepgen-reports-positive-data-phase-1-trial-pgn-edo51-treatment). The FDA placed the clinical development of this compound on hold in December 2024 (https://investors.pepgen.com/news-releases/news-release-details/pepgen-announces-clinical-hold-us-ind-application-initiate). Pepgen has also developed a pPMO for DM1, in which the PMO targets the CUG repeat. The clinical trial is ongoing, but results have not yet been reported (https://investors.pepgen.com/news-releases/news-release-details/pepgen-announces-first-patient-dosed-phase-1-freedom-dm1).

Entrada has developed a circular ‘endosomal escape vehicle’ (EEV) peptide that improves the delivery and efficiency of exon skipping PMOs *in vitro* and *in vivo* in DMD model systems.^46^ An EEV-PMO targeting *DMD* exon 44 is currently being evaluated in patients in a clinical trial. Results from healthy volunteers confirmed exon 44 skipping after a single dose and no hypomagnesemia was reported (https://ir.entradatx.com/news-releases/news-release-details/entrada-therapeutics-reports-positive-preliminary-data-healthy). Based on this technology, Vertex and Entrada are jointly developing an EEV-PMO for DM1, where the PMO targets again the CUG repeats. This compound is currently tested in a clinical trial, but results have not yet been reported.

### Improving muscle uptake

One approach to improve muscle-specific delivery is to conjugate the oligo to an antibody targeting a receptor that is highly expressed on skeletal muscle and heart. The transferrin receptor is currently used as a target. Avidity Biosciences is using an antibody, while Dyne Therapeutics is using a fragment antibody, containing only the antigen-binding domain (Fab).

Preclincial studies showed that using an antibody targeting transferrin receptor 1 could indeed increase delivery of single and doublestranded oligonucleotides to skeletal muscle and heart.^47–49^ Avidity currently has several clinical trials underway. For FSHD and DM1, they are evaluating an antibody-siRNA conjugate targeting *DUX4* and *DMPK,* respectively. For DMD, they are evaluating an antibody-PMO targeting *DMD* exon 44. In FSHD, a significant reduction in *DUX4* target transcripts has been observed in muscle biopsies from treated patients and trends towards functional improvement have been observed intreated patients (https://aviditybiosciences.investorroom.com/2024-06-12-Avidity-Announces-Unprecedented-AOC-1020-Data-from-Phase-1-2-FORTITUDE-TM-Trial-Demonstrating-Greater-Than-50-Percent-Reduction-in-DUX4-Regulated-Genes-and-Trends-of-Functional-Improvement-in-People-Living-with-Facioscapulohumeral-Muscular-Dyst). In DM1 patients, treatment was well tolerated and reduced myotonia and strength was reported. In an open label phase, patients also reported improvements in activities of daily living. A phase 3 clinical trial is currently ongoing (https://aviditybiosciences.investorroom.com/2024-03-04-Avidity-Biosciences-Announces-Positive-AOC-1001-Long-term-Data-Showing-Reversal-of-Disease-Progression-in-People-Living-with-Myotonic-Dystrophy-Type-1-Across-Multiple-Endpoints-Same-Key-Endpoints-Agreed-for-Phase-3-HARBOR-TM-Trial).

In DMD, treatment with 5 mg/kg per month for 3 months resulted in a significant increase in dystrophin expression of 25% compared to baseline (from 7% to 32%) in a biopsy taken after 4 months of treatment (https://aviditybiosciences.investorroom.com/2024-08-09-Avidity-Biosciences-Announces-Positive-AOC-1044-Data-Demonstrated-Significant-Increase-of-25-in-Dystrophin-Production-and-Reduction-of-Creatine-Kinase-Levels-to-Near-Normal-in-People-Living-with-Duchenne-Muscular-Dystrophy-Amenable-to-Exon-44-S). However, 2/9 patients treated with the 5 mg/kg dose discontinued the study due to infusion reactions. Furthermore, a the aforementioned trial with brogodirsen, an unconjugated PMO targeting exon 44, also induced a lare increase in dystrophin, suggesting that part of the high dystrophin increase may be due to the fact that exon 44 skipping is easier to achieve than other dystrophin exons^50^

Preclinical studies with an antibody-fragment linked to ASOs have shown that the fragment antibody can significantly increase delivery to skeletal muscle and heart in a mouse model^51^ of DMD. Dyne is conducting trials in DM1 and DMD and is preparing for trials in FSHD. In DM1 and FSHD they are using an antibody-fragment linked to an RNase H ASO targeting *DMPK* and *DUX4* respectively, while in DMD they are using an antibody-fragment linked to a PMO targeting *DMD* exon 51. Trial results for DM1 reported normalisation of splicing defects, reduced myotonia and increased strength (https://investors.dyne-tx.com/news-releases/news-release-details/dyne-therapeutics-announces-new-clinical-data-achieve-trial-dyne/). For DMD, results show that a 2.fold increase in dose (from 5 to 10 mg/kg/month) results in more than a 3-fold increase in dystrophin restoration (from 0.9% to 3.2%) (https://investors.dyne-tx.com/news-releases/news-release-details/dyne-therapeutics-announces-new-clinical-data-achieve-trial-dyne/). However, using a 20 mg/kg/month dose only slightly increased dystrophin level to 3.7% (https://investors.dyne-tx.com/news-releases/news-release-details/dyne-therapeutics-announces-new-clinical-data-phase-12-deliver/).

Finally, AAV vectors can be used to deliver antisense sequences. This involves engineering the U7 small nuclear ribonuclear protein (U7 snRNP) gene and replacing the original antisense sequence with an antisense sequence of choice. These U7 snRNP genes can then be packaged into an AAV vector to provide an ‘exon skipping gene therapy’ approach, where treatment would require a single dose. Proof of concept in the *mdx* mouse model was already provided over 2 decades ago.^52^ This approach was in development by Audentes, which was later acquired by Astellas, but clinical development was discontinued. Dr Flanigan's group at Center for Gene Therapy Nationwide Children's Hospital has developed an exon 2 skipping U7 snRNP AAV treatment within a clinical setting. There was only enough product to treat 3 patients and theresults presented during conferences suggested mixed efficiencies: less than 10% dystrophin restoration in 2 of the patients, and very high levels of dystrophin (over 80%) in the third patient, who had been treated before the age of 1 (https://www.parentprojectmd.org/nationwide-childrens-hospital-announces-restoration-of-full-length-dystrophin-using-duplication-2-gene-therapy-approach/).

## Future directions

Significant effort has been devoted to improving delivery of ASOs to skeletal muscle and recent reports show encouraging results. These findings generate cautious optimism about achieving sufficient delivery to exert therapeutic effects on muscle diseases. Effectively solving skeletal muscle would pave the way for additional RNA therapies targetting other neuromuscular diseases and broader cohorts of DMD patients, for which companies are already making preparations (in Table 1).

Approaches such as peptide-conjugated morpholino oligomers(pPMOs) and transferrin-receptor targeting also improve delivery to heart tissue in animal models. While this is more challenging to confirm in humans, future, long-term assessments could reveal potential benefits for diseases involving also a cardiac component, such asDM1 and DMD. It should be noted, however, that both DM1 and DMD also include a central nervous system component, which these approaches currently do not address. Although brain also express transferrin receptors, it remains to be confirmed whether the uptake mechanism that appears to be effective in muscle, will also allow uptake through the blood-brain-barrier following systemic administration.

However, even if that were not the case, being able to deliver sufficient ASO amounts to skeletal muscle to prompt therapeutic effects, represents a significant breakthrough that muscle researchers have been aspiring to for decades.
